# Strategies for supplying face masks to the population of Taiwan during the COVID-19 pandemic

**DOI:** 10.1186/s12889-021-11808-3

**Published:** 2021-10-14

**Authors:** Chin-Mei Liu, Charles Tzu-Chi Lee, Shu-Mei Chou, Hai-Yun Ko, Jen-Hsin Wang, Yi-Chien Chih, Chia-Chi Chang

**Affiliations:** 1 Division of Preparedness and Emerging Infectious Diseases, Taiwan Centers for Disease Control, Taipei, Taiwan; 2 Department of Health Promotion and Health Education National Taiwan Normal University, Taipei, Taiwan

**Keywords:** COVID-19 pandemic, Face mask, Face mask supply, Name-based rationing system, Heading: Face mask supply strategies in Taiwan.

## Abstract

**Background:**

The use of face masks has become ubiquitous in Taiwan during the early COVID-19 pandemic. A name-based rationing system was established to enable the population of Taiwan to purchase face masks. This study is to assess the extent and fairness of face mask supply to the public in Taiwan.

**Methods:**

The weekly face marks supplies were collected from name-based rationing system administrative statistics included national health insurance card and e-Mask selling record. National registered population statistics by age, gender, and district were collected from department of statistics ministry of the interior. The number of COVID-19 non-imported cases of Taiwan was collected from Taiwan centers of disease control.

**Results:**

A total of 146,831,844 person times purchase records from February 6, 2020, to July 19, 2020, the weekly average face mask supply is 0.5 mask (per person) at the start of name-based rationing system, and gradually expanded to the maximum 5.1 masks (per person). Comparing the highest weekly total face mask supply (from Apr 9, 2020, to Apr 15, 2020) in aged 0–9 -, 10–19 -, 20–29 -, 30–39 -, 40–49 -, 50–59 -, 60–69 -,70–79 -, 80–89 -, 90–99, and > 100 years to the register population showed similar distribution between mask supplied people and total population (all standardized difference < 0.1).

**Conclusion:**

The masks supply strategies has gradually escalated the number of face masks for the public, it not only has dominant decreased the barrier of acquiring face mask, but a fair supply for total population use of Taiwan.

**Supplementary Information:**

The online version contains supplementary material available at 10.1186/s12889-021-11808-3.

## Introduction

The coronavirus disease 2019 (COVID-19) outbreak originated in Wuhan, China, in December 2019 [1, 2] and spread globally, with a high death toll. In Taiwan, 81 miles off the coast of mainland China, the first COVID-19 case was confirmed on January 21, 2020 [3]. The COVID-19 outbreak occurred just before the Lunar New Year when the Taiwanese workforce in China was expected to travel home for the holidays. Based on the 2003 SARS experience, the public has shown high anxiety over COVID-19 and increased use of preventive measures against COVID-19 [4]. The use of face masks has become ubiquitous in Taiwan and other Asian countries since the outbreak [5].

The increase in the use of face masks exacerbates the shortage of face masks and keeps prices soaring [6]. Faced with a shortage of face masks, the Central Epidemic Command Center (CECC) banned the export of medical face masks on January 23, 2020, and requisitioned all domestically produced medical face masks on January 31, 2020, for professional use in healthcare settings, for public health, and for use by healthy individuals. Soon thereafter, Taiwan increased the production of medical masks and created a large stockpile [7]. Moreover, to ensure universal access to face masks and to increase fairness and transparency in resource allocation for healthy individuals, a name-based rationing system was implemented [8].

During the early stages of the COVID-19 pandemic, guidelines recommended that symptomatic individuals and healthcare professionals should use face masks; however, discrepancies arose in the use of masks in public settings, [6, 9, 10] mainly due to the limited supplies reserved for professionals and because face masks may not effectively prevent coronavirus infection [5, 11]. However, COVID-19 can be transmitted from asymptomatic individuals or within the incubation period [12–14]. The World Health Organization acknowledges that wearing a mask in public places is helpful in pandemics, as even a partial protective effect can have a major influence on transmission [11, 15, 16]. Current evidence suggests that people infected with the novel human coronavirus (SARS-CoV-2) can transmit the virus whether they have symptoms or not, and the use of masks helped contain the pandemic [9, 11, 17]. Modeling study indicated that 50% of face masks coverage in the public with 50% mask effective roughly halve the effective disease transmission rate, 80% coverage even that is only 20% effective still reduces the effective transmission rate by about one-third [18], another modeling result is that if at least 70% of the residents of New York state use face masks such as surgical masks in the public consistently, and compliance of at least 80%, could lead to the elimination of the pandemic [16].

The number of COVID-19 cases in Taiwan remains low, [19] which may be because of the active containment efforts, including allocation of resources to the population, and the substantial use of face masks by the general public to prevent their respiratory droplets from reaching others during the early phase of the COVID-19 pandemic [19–21]. However, the supply strategies whether adequately provided face masks to the population in time for fighting the COVID-19. This study investigated face mask supply under the name-based rationing system during the early phase of COVID-19 in Taiwan to understand the coverage and fairness of face mask supply.

## Methods

The Institutional Review Board (IRB) of National Taiwan Normal University approved this study (Protocol Number: 202008HS001). Using de-identified data, the informed consent was waived by the same ethics committee that approved the study (National Taiwan Normal University). This study followed the Strengthening the Reporting of Observational Studies in Epidemiology (STROBE) reporting guideline for cohort studies, and all methods were carried out in accordance with relevant guidelines and regulations.

### Name-based rationing system

CECC requisitioned all domestically produced medical masks included medical face masks, surgical masks, N95 respirator masks, but only provided medical face masks for the general population. The name-based rationing system allowed for an on-site purchase plan. Face masks were distributed to 6505 National Health Insurance (NHI) pharmacies or drugstores and district public health centers at the start of the plan(1.0) on February 6, 2020 [8]. Within 7 days, each adult was allowed to buy 2 masks per NHI card at a price of 10 New Taiwan Dollar (NT$) (priced at NT$ 5 each, equivalent to USD 0.17 each), each child was allowed to buy 4 masks per NHI card. Subsequently, each adult was allowed to buy 3 masks per NHI card within 7 days, and an online pre-order system (through the eMask website or the NHI application) was added to the name-based rationing system (2.0) on March 12, 2020. Those who have ordered and paid for the masks can pick them up at convenience stores, drug stores, and supermarkets. This ensures even distribution and is convenient for people, such as office workers and students, who lack the time for on-site purchase [22]. With an increase in medical mask production, mandatory face mask wearing in public areas, such as hospitals, public transportation, or crowded places, had been enforced. Moreover, buying 9 masks every 14 days per NHI card for each adult was allowed; the rationing system was upgraded again on April 9, 2020, to allow the public to preorder masks from kiosks at convenience stores (3.0). These steps were designed for elderly people who do not use the internet.

### Data source, study design, and population

We performed a retrospective longitudinal study using administrative statistics of the name-based rationing system from the Ministry of Health and Welfare. Weekly face mask supply was examined using the administrative statistics of the name-based rationing system, and the face masks control files of National Health Insurance and eMask selling records were included in the data set. All data were de-identified by the NHI and eMask manager. The administrative statistics included the number of people who purchased masks, the number of face masks purchased, the sex and age of the purchasers, and the counties where purchases were made. National registered population statistics by age, gender, and district were collected from the Ministry of the Interior Department of Statistics. The population register was according to the national population statistics of Taiwan up to December 15, 2019. The number of local acquired COVID-19 cases by counties was collected from Taiwan Centers of Disease Control (CDC).

### Statistical analysis

Data on masks purchased from February 6 to July 19, 2020, were used in this study. Face mask supply by week, gender, age, and district was described by total number and mean. The weekly average face mask supply was calculated as the number of masks used per week divided by the total population. We assumed that 0.5 mask was used by aged 0–5 years and ≥ 65 years, and 1 mask were used by aged 6–64 years per day. Subgroups were formed according to age, sex, and districts. Standardized differences (SD) were used to compare the distribution of face masks supplied. An SD < 0.1 indicated a negligible difference in the distribution between the people supplied with masks and the total population [23]. Data analysis was performed using SAS version 9.4 (SAS Institute Inc). GeoDa 1.14.0 was used to map the confirmed cases from January 1 to August 7, 2020, and population by county.

## Results

### Sample description

**Fig. 1:**
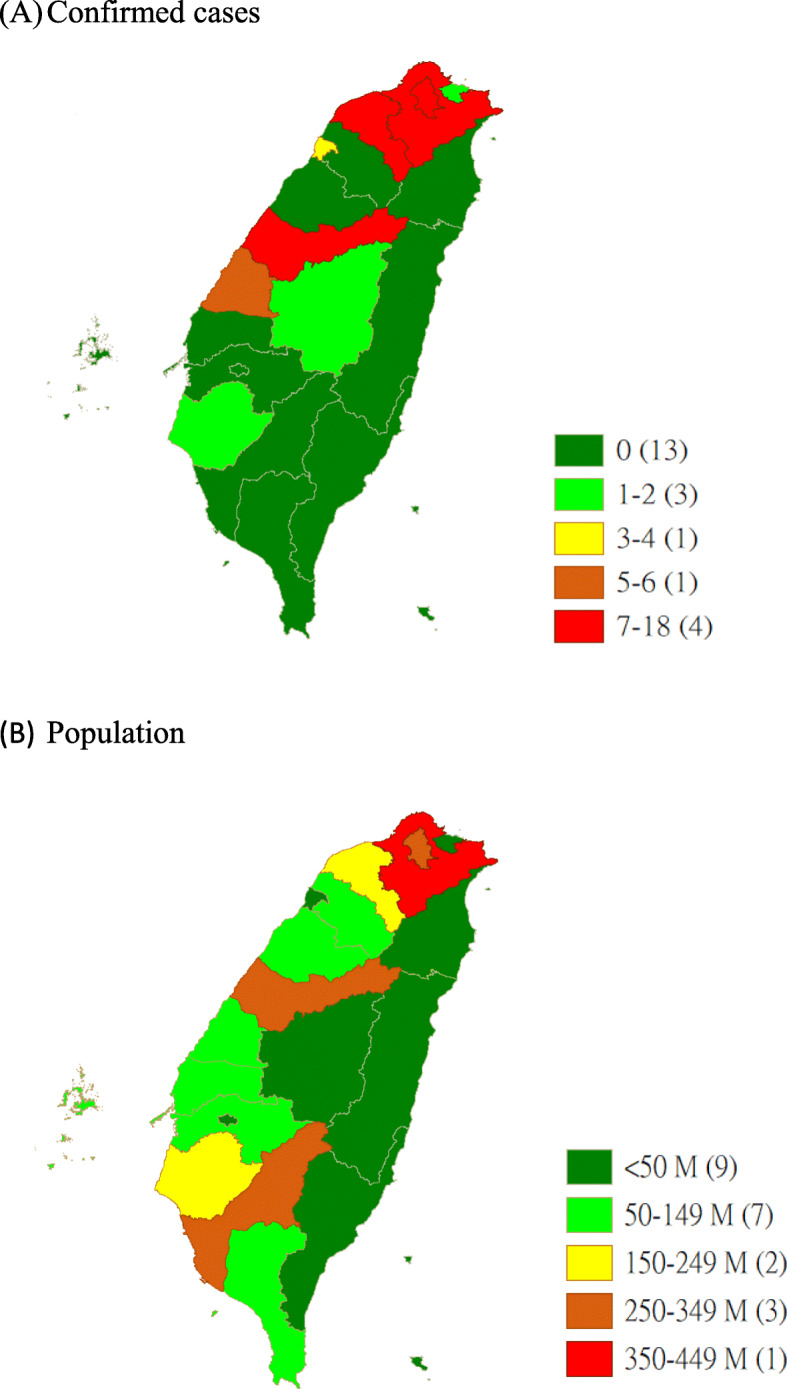
Map of confirmed cases and population by county in Taiwan during COVID-19 pandemic (number of counties in parenthesis)

A total of 55 COVID-19 cases were identified in Taiwan from January 1 to August 7, 2020. The counties and the number of cases are as follows: New Taipei City (18 cases), Taoyuan City (14 cases), Taipei City (8 cases), Taichung City (5 cases), Changhua County (4 cases), Hsinchu City (3 cases), Tainan City (1 case), Keelung City (1 case), and Nantou County (1 case) (Fig. 1). Purchase records from February 6 to July 19, 2020, showed that of 144, 972,457 person-time, 127,881,190 (88.2%) and 17,091,267 (11.8%) were contributed by adults and children, respectively. The weekly maximum purchase records included 11,854,254 person-time and 107,623,594 masks, from April 9 to April 15, 2020. The monthly maximum purchase records included 22,778,511 (27.8%) person-time from April 2 to April 29, 2020. (Table 1).

**Table 1:** Face mask supply and population distribution in Taiwan by month

Period	Age group	Mask supply by person (%)		Register population (%)		Standardized difference
		n	%	n	%	
2/6/2020 to 7/19/2020	0–9	1,728,403	8.66	1,826,052	7.74	0.03
	10–19	1,888,625	9.46	2,132,859	9.04	0.01
	20–59	11,613,738	58.20	14,024,290	59.42	0.02
	≧60	4,725,083	23.68	5,619,920	23.81	0.00
2/6/2020 to 3/4/2020	0–9	1,355,889	10.72	1,826,052	7.74	0.10
	10–19	1,299,456	10.27	2,132,859	9.04	0.04
	20–59	6,886,729	54.43	14,024,290	59.42	0.10
	≧60	3,110,780	24.59	5,619,920	23.81	0.02
3/5/2020 to 4/1/2020	0–9	1,473,339	10.05	1,826,052	7.74	0.08
	10–19	1,479,717	10.09	2,132,859	9.04	0.04
	20–59	8,062,801	55.00	14,024,290	59.42	0.09
	≧60	3,643,267	24.85	5,619,920	23.81	0.02
4/2/2020 to 4/29/2020	0–9	1,421,754	9.01	1,826,052	7.74	0.05
	10–19	1,587,403	10.06	2,132,859	9.04	0.03
	20–59	8,709,093	55.18	14,024,290	59.42	0.09
	≧60	4,063,760	25.75	5,619,920	23.81	0.04
4/30/2020 to 5/27/2020	0–9	931,482	8.04	1,826,052	7.74	0.01
	10–19	1,199,559	10.36	2,132,859	9.04	0.04
	20–59	6,370,606	55.01	14,024,290	59.42	0.09
	≧60	3,079,272	26.59	5,619,920	23.81	0.06
5/28/2020 to 6/24/2020	0–9	798,973	8.90	1,826,052	7.74	0.04
	10–19	938,709	10.45	2,132,859	9.04	0.05
	20–59	4,836,376	53.86	14,024,290	59.42	0.11
	≧60	2,405,910	26.79	5,619,920	23.81	0.07
6/25/2020 to 7/19/2020	0–9	696,351	9.17	1,826,052	7.74	0.05
	10–19	787,826	10.38	2,132,859	9.04	0.05
	20–59	4,069,870	53.61	14,024,290	59.42	0.12
	≧60	2,037,319	26.84	5,619,920	23.81	0.07

Mask supply statistics according to the purchase records of pharmacies, district public health centers, and face masks files of national health insurance

The population register is according to the national population statistics of Taiwan up to Dec 15, 2019

### Face mask supply

**Fig. 2:**
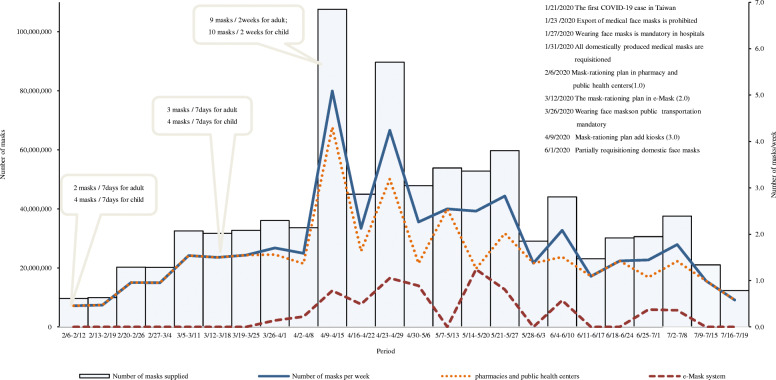
The number of medical masks supplied to the general population weekly during the COVID-19 pandemic in Taiwan

The weekly average face mask supply was 0.5 mask per person at the start of the name-based rationing system, which increased to a maximum of 5.1 masks per person from April 9 to April 15, 2020, and then gradually decreased (Fig. 2) in pharmacies, district public health centers, and electronic platforms (eMask) during the COVID-19 pandemic. In pharmacies and district public health centers, the weekly average face mask supply was 0.5 mask per person at the start of the name-based rationing system, which expanded to a maximum of 4.3 masks per person from April 9 to April 15, 2020, and then gradually decreased. However, the weekly face mask supply in electronic platforms (eMask) was unsteady, with a weekly average of 0.1–1.2 masks per person, and the highest weekly average was 1.2 masks per person from May 14 to May 20, 2020.

### Face mask coverage and fairness

Comparing monthly total face mask supply in 0–9,10-19,20–59 and ≥ 60 years age groups registered in Taiwan, excluding data from the first month after the policy was implemented (February 6 to March 4, 2020), showed a similar distribution of masks supplied in the 4 age groups to that to the total population (SD < 0.1) (Table 1). Subgroup analyses of purchase records from pharmacies and district public health centers and the eMask system for the highest-supply week (April 9 to April 15, 2020) revealed that the total number, number of male, and number of female showed no difference between the people supplied with masks and the total population across 0–9, 10–19, 20–29, 30–39, 40–49, 50–59, 60–69, 70–79, 80–89, 90–99, and > 100 years age groups (SD < 1) (Tables 2, 3 and 4). In urban all age groups showed a similar mask supply distribution to that to the total population (SD < 0.1) (Table 5). In rural districts, excluding the 20–29 years age group, all other age groups showed a similar mask supply distribution to the total population (SD < 0.1) (Table 6). Data from April 23 to April 29, 2020, are presented with results from April 9 to April 15, 2020, in Supplementary Table 1.

**Table 2:** Face mask weekly supply per person and population distribution in Taiwan from April 9 to April 15, 2020 (Total)

Age group	Mask supply		Register population		Standardized difference
	n	%	n	%	
0–9	805,169	6.79	1,995,123	8.45	0.06
10–19	1,082,366	9.13	2,269,369	9.61	0.02
20–29	1,328,407	11.21	3,123,559	13.23	0.06
30–39	1,744,557	14.72	3,559,077	15.08	0.01
40–49	1,956,109	16.50	3,749,616	15.89	0.02
50–59	1,910,911	16.12	3,641,731	15.43	0.02
60–69	1,756,775	14.82	3,037,036	12.87	0.06
70–79	848,608	7.16	1,409,800	5.97	0.05
80–89	353,747	2.98	677,279	2.87	0.01
90–99	65,687	0.55	136,725	0.58	0.00
≥100	1947	0.02	3806	0.02	0.00

Mask supply statistics according to the purchase records of pharmacies, district public health centers, and e-Mask system, and face masks files of national health insurance

The population register is according to the national population statistics of Taiwan up to Dec 15, 2019

**Table 3:** Male groups

Age group	Mask supply		Register population		Standardized difference
	n	%	n	%	
0–9	419,679	7.58	1,033,943	8.83	0.05
10–19	563,525	10.18	1,184,864	10.12	0.00
20–29	637,206	11.51	1,622,236	13.86	0.07
30–39	791,929	14.31	1,785,423	15.25	0.03
40–49	882,420	15.94	1,842,719	15.74	0.01
50–59	880,460	15.91	1,788,072	15.28	0.02
60–69	799,803	14.45	1,456,345	12.44	0.06
70–79	383,894	6.94	644,020	5.50	0.06
80–89	147,170	2.66	284,539	2.43	0.01
90–99	28,435	0.51	61,338	0.52	0.00
≥100	802	0.01	1687	0.01	0.00

Mask supply statistics according to the purchase records of pharmacies, district public health centers, and e-Mask system, and face masks files of national health insurance

The population register is according to the national population statistics of Taiwan up to Dec 15, 2019

**Table 4:** Female groups

Age group	Mask supply		Register population		Standardized difference
	n	%	n	%	
0–9	385,490	6.10	961,180	8.08	0.08
10–19	518,841	8.21	1,084,505	9.12	0.03
20–29	691,201	10.94	1,501,323	12.62	0.05
30–39	952,628	15.08	1,773,654	14.91	0.00
40–49	1,073,689	16.99	1,906,897	16.03	0.03
50–59	1,030,451	16.31	1,853,659	15.58	0.02
60–69	956,972	15.14	1,580,691	13.29	0.05
70–79	464,714	7.35	765,780	6.44	0.04
80–89	206,577	3.27	392,740	3.30	0.00
90–99	37,252	0.59	75,387	0.63	0.01
≥100	1145	0.02	2119	0.02	0.00

Mask supply statistics according to the purchase records of pharmacies, district public health centers, and e-Mask system, and face masks files of national health insurance

The population register is according to the national population statistics of Taiwan up to Dec 15, 2019

**Table 5:** In urban districts

Age group	Mask supply		Register population		Standardized difference
	n	%	n	%	
0–9	549,022	6.55	1,428,536	8.72	0.08
10–19	739,980	8.82	1,556,287	9.50	0.02
20–29	971,109	11.58	2,135,751	13.04	0.04
30–39	1,267,817	15.11	2,519,166	15.38	0.01
40–49	1,411,139	16.82	2,644,765	16.14	0.02
50–59	1,357,552	16.18	2,519,040	15.38	0.02
60–69	1,241,407	14.80	2,123,367	12.96	0.05
70–79	576,794	6.88	946,921	5.78	0.05
80–89	226,685	2.70	416,334	2.54	0.01
90–99	45,298	0.54	89,317	0.55	0.00
≥100	1394	0.02	2655	0.02	0.00

Mask supply statistics according to the purchase records of pharmacies, district public health centers, and e-Mask system, and face masks files of national health insurance

The population register is according to the national population statistics of Taiwan up to Dec 15, 2019

**Table 6:** In rural districts

Age group	Mask supply		Register population		Standardized difference
	n	%	n	%	
0–9	256,147	7.39	566,587	7.85	0.02
10–19	342,386	9.88	713,082	9.88	0.00
20–29	357,298	10.31	987,808	13.68	0.10
30–39	476,740	13.75	1,039,911	14.40	0.02
40–49	544,970	15.72	1,104,851	15.30	0.01
50–59	553,359	15.96	1,122,691	15.55	0.01
60–69	515,368	14.87	913,669	12.65	0.06
70–79	271,814	7.84	462,879	6.41	0.06
80–89	127,062	3.67	260,945	3.61	0.00
90–99	20,389	0.59	47,408	0.66	0.01
≥100	553	0.02	1151	0.02	0.00

Mask supply statistics according to the purchase records of pharmacies, district public health centers, and e-Mask system, and face masks files of national health insurance

The population register is according to the national population statistics of Taiwan up to Dec 15, 2019

## Discussion

This retrospective longitudinal study showed mask supply coverage and fairness using the administrative statistics of a name-based rationing system. By increasing the production of face masks, the weekly average face mask supply increased to a maximum of 5.1 masks per person. The weekly, monthly, and total face mask supply showed that the name-based rationing system supplied masks fairly to the total population, can provide universal coverage, and was sufficient for daily use for the population.

As COVID-19 cases continued to surge in Wuhan, China, on January 21, 2020, the CECC announced the first confirmed imported case of an individual who resided in southern Taiwan and worked in Wuhan. The Taiwanese public started panic buying and hoarding face masks, [24] and face masks were difficult to find online and offline. Because China produces half of the world’s face masks, a major disruption of the global supply chain occurred. Therefore, Taiwan CDC began distributing surgical masks from the stockpile to convenience stores for purchase by the public [19]. A total of two million surgical masks have been released for sale from January 22 to January 30, 2020. However, a huge gap remains between demand and supply, and panic buying and soaring prices have increased. Healthcare professionals have been the most affected [25].

Because approximately 90% of face masks are imported into Taiwan, [26] the Taiwanese government had to domestic private manufacturers produce considerable medical masks every day, and requisitioned all produced medical face masks, also managed distribution and controlled all supplies during the pandemic The daily production of face mask manufacturers in Taiwan before the outbreak was 1.88 million face masks with a maximum production capacity of 19 million face masks per day. In response to precautionary measures recommended for mitigating COVID-19 infection during the early phase, the CECC announced a free supply of masks for healthcare professionals and an affordable price for the public by introducing the name-based rationing system. We believe that offering easy access to simple and cheap face masks can be effective precautionary measures for COVID-19 infection based on Taiwan’s experience in this outbreak [11].

By comparing monthly and weekly face mask supply to the population of Taiwan, the results revealed that the name-based rationing system (1.0/2.0/3.0), using online, offline, and integrated online and offline strategies, showed a fair distribution of face masks to the population. The main reasons of success in fair supply which were face masks supply are sufficient, the masks price very affordable, provided 20,000 sales points, and district public health centers supplied masks in remote areas; thereby removing any barrier to accessibility. We found that when using the electronic platform alone, the number of masks supplied was much lower than when using the on-site purchase in pharmacies and district public health centers; moreover, unfair mask supply in age groups 0–9, 30–39, 40–49, 60–69, 70–79, and 80–89 years. One important reason was the difference in operation design. The eMask system adopts pre-order masks; the public must wait at least 7 days to obtain masks. Conversely, although on-site purchase limits the number of masks purchased in each pharmacy, one can pay for the mask.

Whether face masks mitigate the spread of COVID-19 is debatable [27]. Panic buying in Taiwan originated due to experience with the SARS crisis nearly 20 years ago [21]. However, although there is a lack of evidence on the effectiveness of the face mask policy during the early COVID-19 pandemic, the Taiwanese government enforced the regulation of mask supply. Increasing evidence suggests that using masks can prevent COVID-19. Surgical face masks can prevent the transmission of human coronaviruses and influenza viruses from symptomatic individuals [28]. Studies have demonstrated that universal masking strategies in healthcare systems are associated with a significantly lower rate of SARS-CoV-2 positivity among healthcare workers [29]. A study evaluating state policy for mandatory mask use showed a decline in the COVID-19 growth rate compared with states that did not have this mandate [30]. The results from an epidemic model indicate that only actively finding symptomatic people and their contacts do not decrease transmission number and should be combined with a mask-wearing campaign [31]. Mass masking for source control is a useful and low-cost adjunct to social distancing and hand hygiene during the COVID-19 pandemic [16]. This approach is particularly useful for a pathogen with a relatively common asymptomatic carriage and can effectively mitigate the transmission of COVID-19.

The high transmissibility of the SARS-CoV-2 and the potential of community outbreaks remain a threat to Taiwan’s healthcare system and society. The CECC announced that mask-wearing is mandatory in schools, places of worship, medical and health facilities, public transportation, entertainment venues (KTVs, sports centers, nightclubs, bars, and amusement parks), cinemas and concerts, markets (night markets, shopping malls, and farmers markets), and large social events [32]. More and more counties have recommended wearing masks in public places, its potential for harm increased face masks and medical waste in the environment, and emerging challenges in solid waste management during and after the pandemic. Thus, it is necessary to properly plan waste management and abandon the single-use face mask, with into reuse masks such as cloth masks to reduce the plastics waste environment in Taiwan [33]. The major strength of this study is that it provides statistical data on the use of a face mask nationwide to understand face mask supply coverage and fairness under the name-based rationing system. Studies related to face mask coverage during the COVID-19 pandemic are lacking. Nonetheless, one substantial limitation of this study is that it lacks on-site face mask purchase information related to estimated face mask supply coverage, excluding the period from April 9 to April 15, 2020, and April 23 to April 29, 2020, as some records were not available.

## Conclusions

This study showed that increasing mask production using mask supply strategies gradually increased the availability of face masks to the public, lowered the barrier for acquiring face masks, and ensured fair distribution to the population.

## Supplementary Information

**Additional file 1: Supplement Table 1.** Face mask weekly supply per person and population distribution in Taiwan from Apr 23, 2020 to Apr 29, 2020

## Data Availability

The datasets used and/or analyzed during the current study are available from the corresponding author upon reasonable request.
