# Supplementary material for: Strategies for supplying face masks to the population of Taiwan during the COVID-19 pandemic
Source: BMC Public Health. 2021 Oct 14;21:1854. doi: 10.1186/s12889-021-11808-3 (PMC8514256; doi:10.1186/s12889-021-11808-3)
Supplement: Supplementary file 1 — Additional file 1: Supplement Table 1. Face mask weekly supply per person and population distribution in Taiwan from Apr 23, 2020 to Apr 29, 2020 [file 12889_2021_11808_MOESM1_ESM.docx]

Supplement Table 1 Face mask weekly supply per person and population distribution in Taiwan from Apr 23, 2020 to Apr 29, 2020

1. Total

| Age group | Mask supply | |  | Register population | | Standardized difference |
| --- | --- | --- | --- | --- | --- | --- |
|  | n | % |  | n | % |  |
| 0-9 | 803,963 | 8.15 |  | 1,995,123 | 8.45 | 0.01 |
| 10-19 | 897,414 | 9.10 |  | 2,269,369 | 9.61 | 0.02 |
| 20-29 | 1,078,396 | 10.93 |  | 3,123,559 | 13.23 | 0.07 |
| 30-39 | 1,443,391 | 14.63 |  | 3,559,077 | 15.08 | 0.01 |
| 40-49 | 1,610,606 | 16.32 |  | 3,749,616 | 15.89 | 0.01 |
| 50-59 | 1,541,553 | 15.62 |  | 3,641,731 | 15.43 | 0.01 |
| 60-69 | 1,440,123 | 14.60 |  | 3,037,036 | 12.87 | 0.05 |
| 70-79 | 709,559 | 7.19 |  | 1,409,800 | 5.97 | 0.05 |
| 80-89 | 287,332 | 2.91 |  | 677,279 | 2.87 | 0.00 |
| 90-99 | 52,883 | 0.54 |  | 136,725 | 0.58 | 0.01 |
| ≥100 | 1531 | 0.02 |  | 3,806 | 0.02 | 0.00 |

Mask supply statistics according to the purchase records of pharmacies, district public health centers, and e-Mask system, and face masks control files of national health insurance

The population register is according to the national population statistics of Taiwan up to Dec 15, 2019.

1. Male

| Age group | Mask supply | |  | Register population | | Standardized difference |
| --- | --- | --- | --- | --- | --- | --- |
|  | n | % |  | n | % |  |
| 0-9 | 417,932 | 9.07 |  | 1,033,943 | 8.83 | 0.01 |
| 10-19 | 466,488 | 10.12 |  | 1,184,864 | 10.12 | 0.00 |
| 20-29 | 514,340 | 11.16 |  | 1,622,236 | 13.86 | 0.08 |
| 30-39 | 654,495 | 14.20 |  | 1,785,423 | 15.25 | 0.03 |
| 40-49 | 727,969 | 15.79 |  | 1,842,719 | 15.74 | 0.00 |
| 50-59 | 709,579 | 15.39 |  | 1,788,072 | 15.28 | 0.00 |
| 60-69 | 652,774 | 14.16 |  | 1,456,345 | 12.44 | 0.05 |
| 70-79 | 321,085 | 6.97 |  | 644,020 | 5.50 | 0.06 |
| 80-89 | 120,937 | 2.62 |  | 284,539 | 2.43 | 0.01 |
| 90-99 | 23,245 | 0.50 |  | 61,338 | 0.52 | 0.00 |
| ≥100 | 634 | 0.01 |  | 1,687 | 0.01 | 0.00 |

Mask supply statistics according to the purchase records of pharmacies, district public health centers, and e-Mask system, and face masks control files of national health insurance

The population register is according to the national population statistics of Taiwan up to Dec 15, 2019.

1. Female

| Age group | Mask supply | |  | Register population | | Standardized difference |
| --- | --- | --- | --- | --- | --- | --- |
|  | n | % |  | n | % |  |
| 0-9 | 386,031 | 7.34 |  | 961,180 | 8.08 | 0.03 |
| 10-19 | 430,926 | 8.20 |  | 1,084,505 | 9.12 | 0.03 |
| 20-29 | 564,056 | 10.73 |  | 1,501,323 | 12.62 | 0.06 |
| 30-39 | 788,896 | 15.01 |  | 1,773,654 | 14.91 | 0.00 |
| 40-49 | 882,637 | 16.79 |  | 1,906,897 | 16.03 | 0.02 |
| 50-59 | 831,974 | 15.83 |  | 1,853,659 | 15.58 | 0.01 |
| 60-69 | 787,349 | 14.98 |  | 1,580,691 | 13.29 | 0.05 |
| 70-79 | 388,474 | 7.39 |  | 765,780 | 6.44 | 0.04 |
| 80-89 | 166,395 | 3.17 |  | 392,740 | 3.30 | 0.01 |
| 90-99 | 29,638 | 0.56 |  | 75,387 | 0.63 | 0.01 |
| ≥100 | 897 | 0.02 |  | 2,119 | 0.02 | 0.00 |

Mask supply statistics according to the purchase records of pharmacies, district public health centers, and e-Mask system, and face masks control files of national health insurance

The population register is according to the national population statistics of Taiwan up to Dec 15, 2019.

1. Urban districts

| Age group | Mask supply | |  | Register population | | Standardized difference |
| --- | --- | --- | --- | --- | --- | --- |
|  | n | % |  | n | % |  |
| 0-9 | 568,860 | 8.06 |  | 1,428,536 | 8.72 | 0.02 |
| 10-19 | 620,873 | 8.80 |  | 1,556,287 | 9.50 | 0.02 |
| 20-29 | 798,478 | 11.32 |  | 2,135,751 | 13.04 | 0.05 |
| 30-39 | 1,059,863 | 15.02 |  | 2,519,166 | 15.38 | 0.01 |
| 40-49 | 1,173,257 | 16.63 |  | 2,644,765 | 16.14 | 0.01 |
| 50-59 | 1,103,586 | 15.64 |  | 2,519,040 | 15.38 | 0.01 |
| 60-69 | 1,022,556 | 14.49 |  | 2,123,367 | 12.96 | 0.04 |
| 70-79 | 485,104 | 6.87 |  | 946,921 | 5.78 | 0.04 |
| 80-89 | 185,805 | 2.63 |  | 416,334 | 2.54 | 0.01 |
| 90-99 | 36,811 | 0.52 |  | 89,317 | 0.55 | 0.00 |
| ≥100 | 1119 | 0.02 |  | 2,655 | 0.02 | 0.00 |

Mask supply statistics according to the purchase records of pharmacies, district public health centers, and e-Mask system, and face masks control files of national health insurance

The population register is according to the national population statistics of Taiwan up to Dec 15, 2019.

1. Rural districts

| Age group | Mask supply | |  | Register population | | Standardized difference |
| --- | --- | --- | --- | --- | --- | --- |
|  | n | % |  | n | % |  |
| 0-9 | 235,103 | 8.37 |  | 566,587 | 7.85 | 0.02 |
| 10-19 | 276,541 | 9.84 |  | 713,082 | 9.88 | 0.00 |
| 20-29 | 279,918 | 9.96 |  | 987,808 | 13.68 | 0.12 |
| 30-39 | 383,528 | 13.65 |  | 1,039,911 | 14.40 | 0.02 |
| 40-49 | 437,349 | 15.56 |  | 1,104,851 | 15.30 | 0.01 |
| 50-59 | 437,967 | 15.58 |  | 1,122,691 | 15.55 | 0.00 |
| 60-69 | 417,567 | 14.86 |  | 913,669 | 12.65 | 0.06 |
| 70-79 | 224,455 | 7.99 |  | 462,879 | 6.41 | 0.06 |
| 80-89 | 101,527 | 3.61 |  | 260,945 | 3.61 | 0.00 |
| 90-99 | 16,072 | 0.57 |  | 47,408 | 0.66 | 0.01 |
| ≥100 | 412 | 0.01 |  | 1,151 | 0.02 | 0.00 |

Mask supply statistics according to the purchase records of pharmacies, district public health centers, and e-Mask system, and face masks control files of national health insurance

The population register is according to the national population statistics of Taiwan up to Dec 15, 2019.
